# Pediatric physeal slide-traction plate fixation for pathological distal femoral fracture caused by unicameral bone cyst in adolescents

**DOI:** 10.1186/s12891-020-03526-5

**Published:** 2020-07-29

**Authors:** Jin Li, Saroj Rai, Renhao Ze, Xin Tang, Ruikang Liu, Pan Hong

**Affiliations:** 1 Department of Orthopaedic Surgery, Union Hospital, Tongji Medical College, Huazhong University of Science and Technology, Wuhan, 430022 China; 2 Department of Orthopaedics and Trauma Surgery, National Trauma Center, National Academy of Medical Sciences, Mahankal, Kathmandu, Nepal; 3 First School of Clinical Medicine, Tongji Medical College, Huazhong University of Science and Technology, Wuhan, China

**Keywords:** Unicameral bone cyst, Distal femoral fracture, Pathological fracture, Limb-length difference

## Abstract

**Background:**

Most patients suffering from distal femoral unicameral bone cysts (UBCs) are adolescents that require an early return to normal activities, including school attendance and sports exercises. However, the optimal choice of implants for such patients remains controversial. This study evaluated the application of pediatric physeal slide-traction plate (PPSP) in the treatment of pathological distal femoral fracture caused by UBCs.

**Methods:**

Between Jan 2014 and Jan 2016, 11 (male = 6, female = 5) patients were reviewed retrospectively. Age, sex, operative time, limb-length discrepancy (LLD), and valgus angulation were all recorded for every patient.

**Results:**

The average age of 11 patients was 12.2 ± 1.1 years. The operating time was 94.8 ± 7.8 min. The postoperative hospital stay was 5 to 7 days. The epiphyseal morphology in the operative leg was nearly normal. The plate was removed in an average of 19.5 ± 3.1 months. The knee range of motion (ROM) was normal in 9 patients, whereas 2 female patients reported a loss of less than 10 degrees of ROM as compared to the contralateral knee joint. Breakage of plates or refracture did not occur in our cases. All patients had a follow-up of at least 24 months. At the latest follow-up visit, all patients walked without a limp. None of the patients manifested obvious LLD and valgus deformity.

**Conclusion:**

PPSP combined with curettage and bone grafting allows early mobilization and produces satisfactory outcomes for pathological fracture of distal femur secondary to UBCs in adolescents.

## Background

Unicameral bone cysts (UBC) are the most common benign bone lesions in children. The reported peak age is between 3 and 14 years, with the mean age at the time of diagnosis being 9 years [1–4]. UBC develops mostly in the proximal humerus and proximal femur, and the occurrence in the distal femur is rare. UBCs around the weight-bearing limbs usually require surgical intervention. The indications for surgical intervention are continued pain, impending/recurrent fracture, and prevention of secondary deformity [2, 3, 5].

There are reports on the surgical intervention with debridement and bone grafts without instrumentation for the UBCs in Children [3, 4]. However, we believe that adolescents are usually an active group of populations requiring early return to school and sports activities. Therefore, they need not only the eradication of the lesion but also the robust fixation for stability.

External fixation [6], Kirschner wire (K-wire) [7], elastic stable intramedullary nail (ESIN) [8], and traditional rigid plate fixation [9] have been reported to treat the distal femoral fractures in children. However, an adolescent with a visible physeal growth plate represents a unique group. The pediatric physeal slide-traction plate (PPSP) was proposed to treat comminuted distal femoral fractures in children [10]. It is routinely removed at about 8 months after the surgery. In contrast, any pathological fracture requires implant placement for an extended period of time until the lesion heals completely.

This study aims to assess the application of PPSP for pathological fractures of the distal femur caused by UBCs in adolescents.

## Methods

### Patient demographics and assessment

Between Jan 2014 and Jan 2016, 11 patients, including 6 males and 5 females, who underwent PPSP for UBC of the distal femur, were reviewed retrospectively. Inclusion criteria were: (1) patients aged between 10 and 16 years; (2) with pathological fractures of the distal femur secondary to UBCs; (3) having a follow-up period of 24 months or more. Patients older than 16 years, open fractures, and incomplete medical records were excluded from the study. Demographic information, including age, sex, disease diagnosis, average duration from fracture to surgery, operative time, and duration of hospital stay, were recorded from the hospital database. Postoperative data, including limb-length discrepancy (LLD), range of motion (ROM) of the knee joint, valgus angulation, and any complications, were recorded at every follow-up visit. LLD was measured both clinically and radiologically, and after the removal of hardware, the LLD was measured clinically since no more X-ray was mandatory. Valgus angulation was measured on the radiograph (difference between operative mechanical Lateral Distal Femoral Angle (mLDFA) and contralateral mLDFA), but not all patients undertook standing X-ray, and some patients only took supine position X-ray. After the hardware removal, the valgus angulation was measured only clinically. The loss of ROM was measured as the difference of active ROM between operative and contralateral leg.

### Surgical technique and postoperative care

**Fig. 1:**
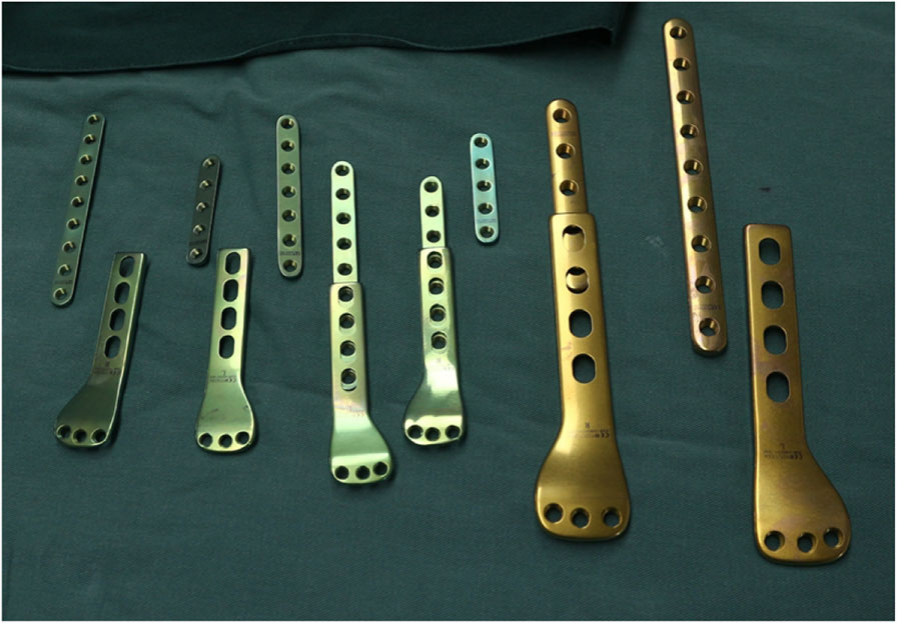
Different sizes of PPSP

PPSP was manufactured by Double Engine Medical Material Co. Ltd. (Xiamen, China), and the plate is divided into two parts (head and body), which allows slide traction (Fig.1).

**Fig. 2:**
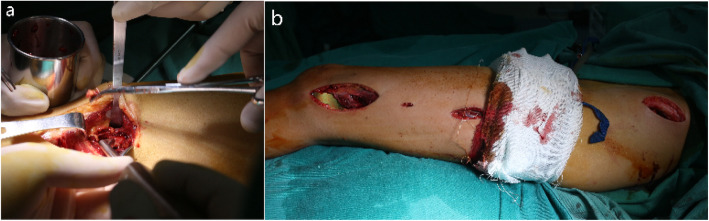
Curettage and osteosynthesis

**Fig. 3:**
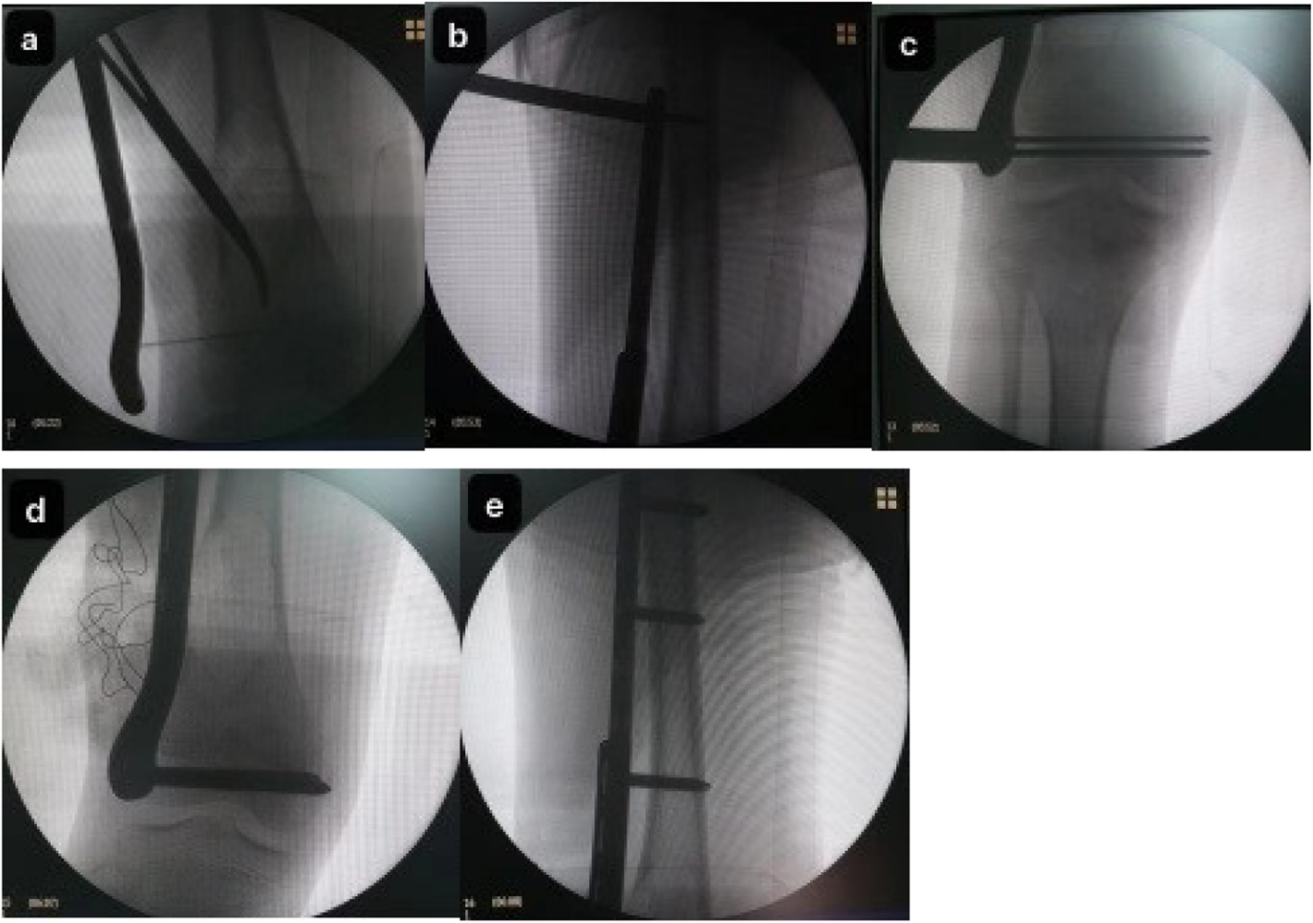
Placement of the PPSP. **a** Identification of the physis. **b** Temporary fixation of the proximal hole of PPSP. **c** Temporary fixation of the distal holes of PPSP. **d** Placement of screws in the distal holes of PPSP. **e** Placement of screws in the proximal holes of PPSP

All the patients underwent surgery under general anaesthesia in the supine position. A pneumatic tourniquet is applied routinely. The lateral approach was used to visualize the fracture site, and the lateral femoral condyle was exposed. The cartilage membrane surrounding the growth plate requires special attention. In most cases, there was no significant fracture displacement. The entire cavity was curetted, and the extracted tissues were sent for histopathological examination (Fig. 2). Fracture margins were freshened. Autologous bone graft was harvested from the ipsilateral iliac crest. The cavity was filled with autologous bone graft and allogeneic bone (Shanxi Auri Biomaterial Co., Ltd). Intraoperative fluoroscopy was performed to ascertain the relative location of the distal holes of the head part and the growth plate. Then, the PPSP was introduced, and slide performance was checked before placing the screw at the proximal hole in the body part. Then the screws for the distal head part were inserted one by one, and 3 more screws were inserted into the holes of the body part (Fig. 3). All the screws were locking screws. After the fixation, the wound was closed in layers. The operated limb was immobilized in a long leg slab postoperatively for 2 weeks to help alleviate the pain.

The suture was removed after 2 weeks. The passive-assisted knee range of motion (ROM) exercise was initiated after 2 weeks as per pain tolerance. Active ROM exercise and partial weight-bearing exercise was initiated after 4 weeks. Then, the patient was followed-up at every 3 months on an out-patient basis.

### Statistical analysis

All descriptive data were analyzed using SPSS and presented as the mean ± SD.

## Results

The average duration from fracture to surgery was 3.5 days. As shown in Table 1, the average age of 11 patients was 12.2 ± 1.1 years (10 years to 14 years). The operating time was 94.8 ± 7.8 min. The postoperative hospital stay was 5 to 7 days. Full-length radiographs of lower extremities at follow-up visits revealed the PPSP could be extended as the femur grew. Besides, the epiphyseal morphology in the operative leg was nearly normal. The plate was removed in an average of 19.5 ± 3.1 months (15 to 24 months) after the surgery. No plate breakage or refracture occurred in our case series. All patients had a follow-up of at least 24 months, and the average follow-up period was 36.5 ± 8.3 months (24 to 47 months).

**Table 1:** Clinical information

No.	Age	Sex	Duration of Surgery (min)	Follow-up (M)	LLD (mm)	Plate Removal(M)	Valgus Angulation (Degree)
1	13	m	99	36	4	23	2
2	14	f	103	31	2	19	0
3	12	m	93	43	3	21	4
4	12	f	88	41	2	18	4
5	13	m	87	24	2	15	6
6	11	f	76	44	4	24	4
7	13	m	97	47	4	17	3
8^a^	12	f	99	48	2	24	5
9	10	m	101	31	3	24	6
10	13	m	99	24	4	18	6
11	11	f	101	32	2	15	4

*Min* minutes; *M* months; *LLD* limb length differences; *mm* millimeter

LLD and valgus angulation was recorded at last follow-up visit

**Fig. 4:**
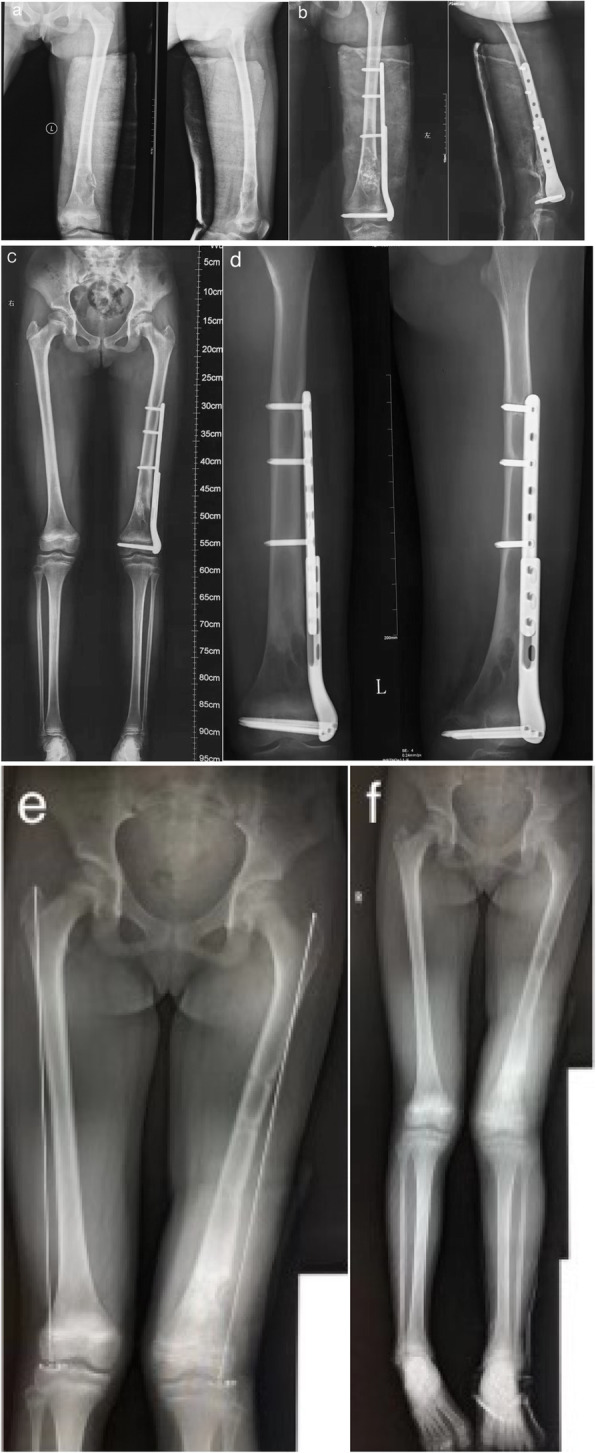
A 12-year-old girl was diagnosed with distal femoral fracture secondary to the unicameral bone cyst and treated with PPSP and bone graft. **a** Preoperative radiographs on AP and lateral view. **b** Postoperative radiographs on AP and lateral view. **c** .Full-length radiograph of lower extremities at 17-month. **d** AP and lateral radiographs of the femur at 22-month. **e** AP radiograph of the femur after the plate removal at 25-month. **f** Full-length radiograph of lower extremities after the plate removal at 25-month

^a^example patient in Fig. 4

The immediate postoperative period was uneventful. At 6-month postoperatively, all patients were able to walk normally. At the latest follow-up visit, all patients walked without a limp. The knee ROM was normal in 9 patients. However. 2 female patients reported a small loss of flexion (< 10 degrees). None of the patients manifested obvious LLD, and the average LLD is 2.9 ± 0.9 mm. The average valgus angulation is 4 ± 1.4 degrees, and no obvious genu valgum was observed during the follow-up.

Figure 4 (Patient No. 8 in Table 1) shows that a 12-year-old girl suffered a distal femoral fracture secondary to UBC, and was treated with open reduction and internal fixation (ORIF) with PPSP and bone grafting

## Discussion

PPSP proved to be a feasible choice for pathological fracture of the distal femur caused by UBCs in adolescents without significant complications, including LLD and valgus angulation.

The ideal treatment for UBC lesion in the distal femur includes sufficient curettage and bone grafting, accurate reduction of the fractures, and stable fixation without disturbing normal growth. Traditional implants, including K-wire, screws and ESIN, are not able to provide adequate stability for pathological distal femoral fractures in teenagers. The high rate of pin tract infection (PTI) and its cumbersomeness prevents external fixator from being the ideal choice. Traditional plate with appropriate bending might be used for this situation. However, the stripping of periosteum around the physeal growth plate is inevitable, leading to the reduced blood supply and growth arrest resulting in valgus deformity [11]. Even if the rigid plate is placed bridging the growth plate without screws penetration, the implant needs to be removed early to allow normal growth.

To overcome these drawbacks, the PPSP system was adopted at our institute. The PPSP system was proposed by Lin et al. [10], for comminuted distal femur fractures in children. The mean removal time after the surgery in their study was 8.4 months. But in our study, we did not remove the plate until complete healing of the UBCs, and the average duration was 19.5 ± 3.1 months. Although the implant removal time was much longer, no significant complications, including LLD and valgus angulation, were noticed in our study, possibly due to the appropriate placement of PPSP. The PPSP is a locking plate in essence, and in order to maintain normal sliding, there is no need for close contact between the plate and bony surface. The lateral aspect of the distal femur was merely minimally stripped, and the compression of the plate against the periosteum is quite limited.

The etiology of UBCs remains controversial, and blockage in the venous drainage remains a favored mechanism among authors [12, 13]. Therefore, decompression techniques using needles, curette, or implants, including cannulated screws, K-wires, or ESIN, have been reported, but the recurrence rate varied from 15 to 88% [2, 14, 15]. However, the correct and prolonged application of PPSP resulted in a satisfactory outcome for UBCs treatment in the distal femur. Partial recurrence did not result in pathological fracture with the support of PPSP, and an additional bone grafting procedure would suffice.

UBC occur commonly in the adolescence, and pathological fracture, especially in the lower extremity, is a serious problem requiring surgical intervention. For unintentional findings of UBCs without fractures, prevention of fracture and pain management are preferred treatment [4]. Less aggressive methods, including injection of steroids [16], bone marrow [17], and demineralized bone matrix (DBM) [18], have been reported for UBCs treatment. In younger patients, UBCs around distal femur was treated with curettage, bone grafting followed by immobilization with a slab, because these group of patients exhibits faster healing. However, teenager is a special group, usually requiring an early return to school and sports activities. Therefore, besides curettage and bone grafting, internal fixation is required for earlier mobilization [19]. Internal fixation combined with curettage has been reported better clinical outcomes for pathological fractures [20, 21].

At our institute, adolescents with distal femoral fractures secondary to UBCs are managed by ORIF, curettage, and bone grafting. To the best of our knowledge, this study is the first to report the utilization of PPSP in pathological fractures in the distal femur. All the patients showed satisfactory clinical outcomes with a complete or partial resolution of UBCs following surgery. Although no serious LLD or valgus angulation was seen in our study, minimal ROM loss was evident in two female patients, partly because of their reluctance to undergo ROM exercise.

There were several limitations in our study. Firstly, the sample size was small because of the rarity of UBC at the distal femur. Secondly, all patients were not followed-up until adulthood, and the potential influence of PPSP on the growth is not thoroughly investigated. A multicenter and prospective study with a large sample size and longer follow-up might produce a more convincing conclusion.

## Conclusion

PPSP combined with curettage and bone grafting allows early mobilization and produces satisfactory outcomes for pathological distal femoral fractures secondary to UBCs in adolescents.

## Data Availability

The datasets supporting the conclusion of this article are included within the article. Upon request, raw data can be provided by the corresponding author.

## Abbreviations

UBC: Unicameral bone cyst
PPSP: Pediatric physeal slide-traction plate
LLD: Limb-length discrepancy
ROM: Range of motion
K-wire: Kirschner wire
ESIN: Elastic stable intramedullary nail
mLDFA: mechanical Lateral Distal Femoral Angle
ORIF: Open reduction and internal fixation
PTI: Pin tract infection
DBM: Demineralized bone matrix
